# Ultrasonically and Iontophoretically Enhanced Drug-Delivery System Based on Dissolving Microneedle Patches

**DOI:** 10.1038/s41598-020-58822-w

**Published:** 2020-02-06

**Authors:** Moonjeong Bok, Zhi-Jun Zhao, Sohee Jeon, Jun-Ho Jeong, Eunju Lim

**Affiliations:** 1 Department of Science Education/Creative Convergent Manufacturing Engineering, Dankook University, Yongin, 16890 South Korea; 2 Nano-Convergence Mechanical Systems Research Division, Korea Institute of Machinery and Materials, Daejeon, 34103 South Korea; 3 Department of Nano Mechatronics, University of Science and Technology, Daejeon, 34103 South Korea

**Keywords:** Microbiology techniques, Drug delivery

## Abstract

A multifunctional system comprised of hyaluronic acid microneedles was developed as an effective transdermal delivery platform for rapid local delivery. The microneedles can regulate the filling amount on the tip, by controlling the concentration of hyaluronic acid solution. Ultrasonication induces dissolution of the HA microneedles via vibration of acoustic pressure, and AC iontophoresis improves the electrostatic force-driven diffusion of HA ions and rhodamine B. The effect of ultrasound on rhodamine release was analyzed *in vitro* using a gelatin hydrogel. The frequency and voltage dependence of the AC on the ion induction transfer was also evaluated experimentally. The results showed that the permeability of the material acts as a key material property. The delivery system based on ultrasonication and iontophoresis in microneedles increases permeation, thus resulting in shorter initial delivery time than that required by delivery systems based on passive or ultrasonication alone. This study highlights the significance of the combination between ultrasonic waves and iontophoresis for improving the efficiency of the microneedles, by shortening the reaction duration. We anticipate that this system can be extended to macromolecular and dependence delivery, based on drug response time.

## Introduction

Transdermal drug delivery system enables the painless and sustainable release of drugs when compared to traditional drug delivery options, such as injection formulations and oral delivery. In the delivery of physiologically active substances, the main issue is achieving the desired amount of absorption and permeation enhancement due to the low permeability of the stratum corneum, the outer layer of the skin. Thus, there is a need for transdermal drug delivery systems that can inject the required amount of drug and increase the rate of drug release. Recently, various physical promoters have been examined to improve transdermal drug delivery, e.g., microneedles, iontophoresis, and ultrasound^1–4^. This is a general strategy for performing transdermal drug delivery.

One of these methods, microneedles, is a minimally invasive method of drug delivery, where the needle length is less than 1000 µm. It can effectively deliver drugs or active ingredients to the skin by crossing over the stratum corneum^5–11^. Among the various types of microneedles^5–11^, dissolving microneedles are made of a water-soluble polymer that exhibits excellent biocompatibility. The dissolving microneedles penetrate the skin and get dissolved by the fluid in the skin to deliver drugs or effective ingredients^8,10,11^. On the other hand, the cavitation phenomenon is applied during ultrasound-mediated drug delivery. Ultrasound causes micro-vibrations in the liquid and solid mediums which increase the kinetic energy of the molecules; cavitation phenomenon accompanies the generation and loss of bubbles due to the pressure change caused by ultrasonic energy^12,13^. Conventional ultrasound has been used to enhance the absorption of drugs^14–16^, DNA/RNA^17,18^, genes^19,20^, and other compounds into cells^21^ and tissues^22^. In the case of skin, when ultrasound energy meets the microbubbles existing at the skin interface, contraction, relaxation, vibration, and acoustic storms are generated^23–27^. Another phenomenon in ultrasound-mediated drug delivery is thermal effects, where energy absorption in the membrane due to viscous friction^23^, and collapses of bubbles in the liquid medium^12,13^.

Iontophoresis (IP), is a non-invasive technique that increases the penetration of ionized compounds into the skin, in the presence of an electric field. The ionized material is also introduced into the tissue by electrical shock and electro-osmosis^28–30^. This method has the potential to control drug penetration by varying the density and time of the applied current. Because of its easy application, non-invasiveness, and increased drug penetration into target tissues, it has been used to deliver macromolecules, such as proteins^31,32^ and peptide dendrimers^33,34^.

To further improve the exact amount of drug that is delivered as well as the efficacy of drug delivery, we proposed a multi-functional hyaluronic acid (HA) microneedle system combined with ultrasound aided iontophoresis effect. To date, a multi-strategy approach to transdermal delivery in dissolving microneedles has been rare. HA, which is the major component of the dissolving microneedle, is a natural water-soluble polymer with excellent biocompatibility^35–38^ and enables dissolution of the microneedles. HA is a binding agent approved by the US food and Drug Administration (FDA) and is widely used in the beauty and healthcare industry^35,37^. To fabricate the controlled filling amount of HA microneedles, we compared the different concentrations. To observe the dissolution phenomenon of the prepared microneedle, gelatin hydrogel was used. Gelatin contains a large amount of water and is easy to use as a tissue model for *in vitro* analysis. In addition, the gelatin acts as cavitation source and medium to transmit ultrasonic energy^22^. To enhance transdermal transport, 1.7 MHz ultrasound was used, and frequency and voltage dependencies were evaluated for alternating IP. HA is biopolymer with negatively charged ions^36–38^, and rhodamine B was used as a positively charged drug model^39,40^. The difference in the release of rhodamine was analyzed, and the fluxes were compared in terms of transport. Thus, we demonstrated the enhancement of the permeability in dissolving microneedles through the combined system of ultrasonication and iontophoresis and the increase in drug release in the early stages. These studies provide a basis for further development of optimized drug response time, drug amount control in microneedles.

## Results and Discussion

### Combination of ultrasound and electric pulses with HA microneedle dissolution in hydrogel

**Figure 1:**
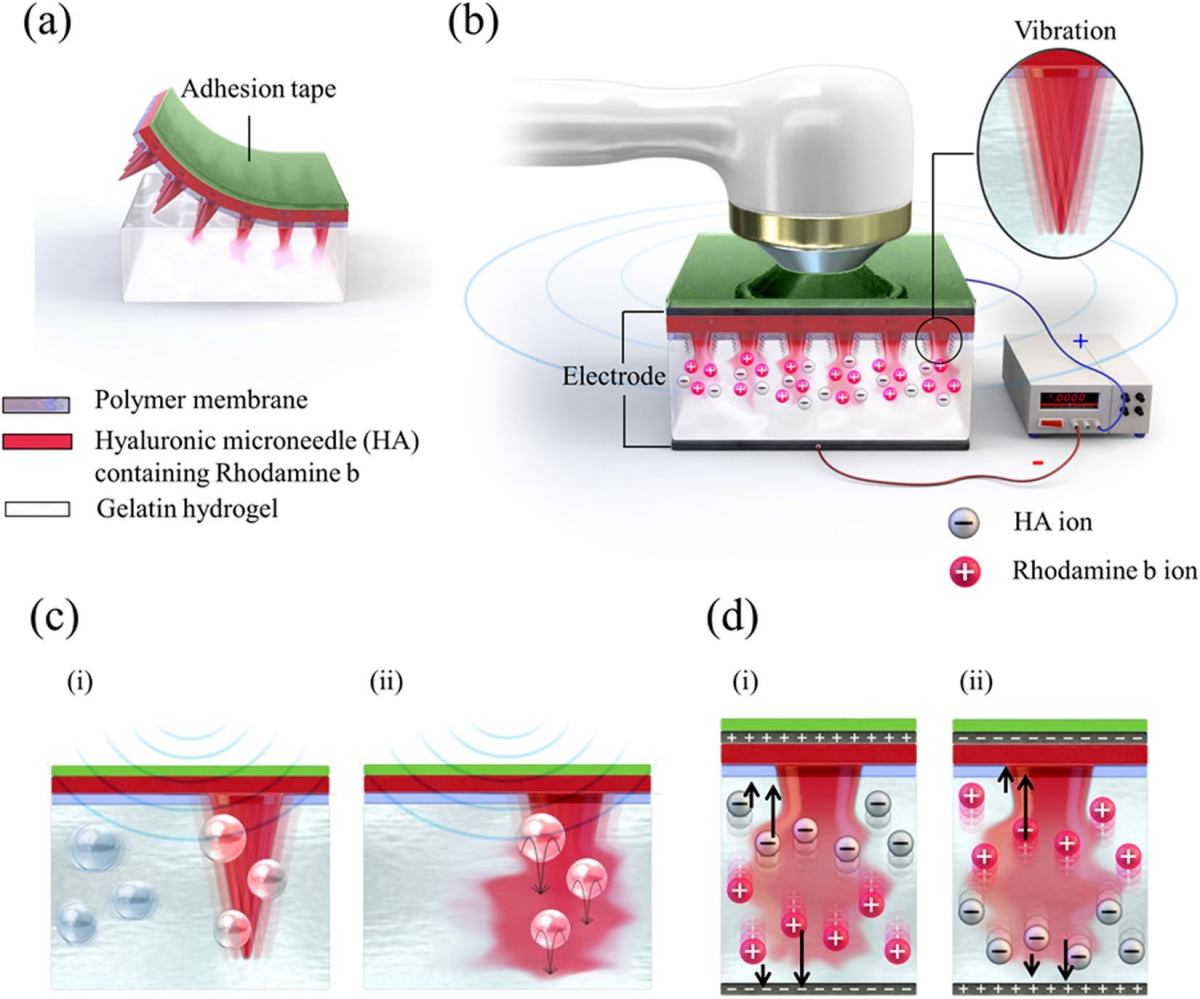
Schematic illustration of (**a**) the attachment structure of HA microneedles in gelatin hydrogel, (**b**) multi-system structure of HA microneedles, and the ultrasonic and electric field in gelatin hydrogel. The inset image indicates the vibration of the needle. Schematic representation of the predicted scenario of (**c**) the dissolution mechanism using ultrasound; (i) fine bubbles and the vibration of the needles by ultrasound and (ii) needle dissolution by ultrasound, (d) The predicted dissolution mechanism and ion direction in relation to the direction of the electric field; (i) voltage > 0 and (ii) voltage < 0.

Figure 1a schematically illustrates the dissolution of hyaluronic acid (HA) microneedles, which are water-soluble polymers of disaccharides, in the body^35–38^. The optically transparent gelatin hydrogel was used as a tissue model, making it easy to observe the dissolution of HA microneedles. The polymer membrane on the hydrogel replicates the waterless stratum corneum and acts as a diffusion barrier to prevent the sudden bulk dissolution of HA microneedles. The device used to increase the dissolution rate of HA microneedles is shown in Fig. 1b. There were two sources for stimulating dissolution, namely ultrasonic stimulation, and continuous electric field. Adhesive tape was used to prevent dissolution from the impedance matching gel during ultrasonic stimulation. To apply an electric field, an aluminum foil was placed under the hydrogel, and a silver paste was used as an electrode. The system consisted of three functional parts. First; the drug-intercalated HA microneedles, which penetrate the gelatin to form holes and deliver the drug. Second; the ultrasonic waves cause the needles to vibrate and jet the drug in the gel of cavitation source^22^. Third; a continuous electric field, which electrically repulses the ionized compound.

### Effect of ultrasonic stimulation on HA microneedle dissolution

In the case of using ultrasound for transdermal delivery, the mechanism shown in Fig. 1c can be predicted to occur. In gelatin gel of a tissue mimicking material, bubbles or other defects exist in the gel as cavitation source during gelation^22^, thus the cavitation can be collapsed by sound pressure (vibration) of ultrasonic waves with a specific frequency^23–27^, resulting in a microjet due to bubble collapse at the needle portion. Thus, physical forces can be generated to increase the effectiveness of drug delivery in gelatin by causing permeabilization.

### Effect of continuous electric field on HA microneedle dissolution

The effect of the continuous sine wave of iontophoresis on HA microneedle dissolution is shown in Fig. 1d. The voltage was continuously applied to the anode and the cathode. The HA microneedles consist of ionic materials, namely, negatively charged HA^36–38^ and positively charged rhodamine B^39,40^. As shown in Fig. 1d, when positive voltage was applied on the microneedles, positively charged rhodamine B was pushed to the tip, and negatively charged HA was pushed towards the base of the needle due to electric repulsion, and vice versa, when a negative voltage was applied. Thus, the ionic materials accelerated the dissolution under the applied electric field.

### Fabrication of HA microneedles

**Figure 2:**
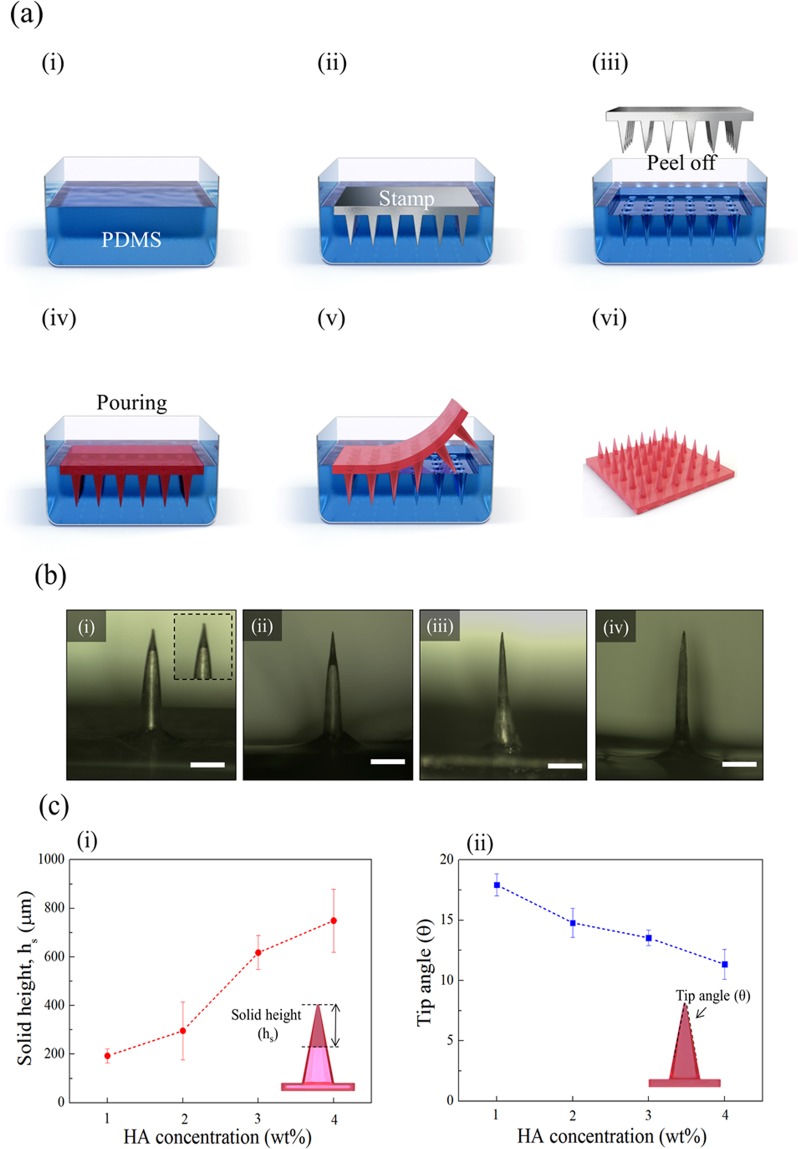
(**a**) HA microneedle array mold fabrication process; (i) pouring of PDMS, (ii) placing the microneedle stamp on the PDMS solution, (iii) curing and removing the stamp from PDMS mold, (iv) pouring the prepared HA solution onto the mold, (v) drying the HA solution, and (vi) the completed microneedle array. (**b**) Optical images of the fabricated microneedle at the concentrations of different HA solution; (i) 1 wt%, (ii) 2 wt%, (iii) 3 wt%, and (iv) 4 wt%. (Scale bar = 300 μm) (**c**) Comparison of microneedle properties at different HA concentrations; (i) solid height (hs) and (ii) tip angle.

The fabrication of the HA microneedles consisted of two parts (Fig. 2a), namely the fabrication of the mold (Fig. 2a(i–iii)), and the fabrication of the microneedle patch (Fig. 2a(iv–vi). In the fabrication of the mold, the microneedle stamp was used to fabricate negative molds with polydimethylsiloxane (PDMS) via the molding technique. The stamp was placed on the poured PDMS, which was then cured in an oven, and the stamp was peeled off. The HA solution of each concentration was poured in the same way to make a microneedle patch with the completed mold and then dried in an oven. The experimental details are provided in the experimental section. Figure 2a(vi) shows a patch of the completed HA microneedles. Figure S1 shows the scanning electron microscopy image of the HA microneedle patch. The distance between needles is 2000 μm, and the needle height is 1000 μm. The needle shape as measured by the solid height (hs) and tip angle (θ) varied according to HA concentration and was evaluated using the optical image. Figure 2b shows the optical images of the microneedles at different HA concentrations. The solid height refers to the height of the portion where the HA is filled in the needle. The lower the concentration of HA solution in the needle, the hollower the needle structure was. As the HA solution concentration increased, the solid height increased (Fig. 2c(i)). Besides, the tip angle of the needle decreased with increasing the concentration of HA solution (Fig. 2c(ii)). Therefore, it was confirmed that the inside of the needle was filled as the concentration of HA solution increased, and it was found that the injected amount could be controlled.

### Observation of the dissolution phenomenon and mechanism in hydrogel

**Figure 3:**
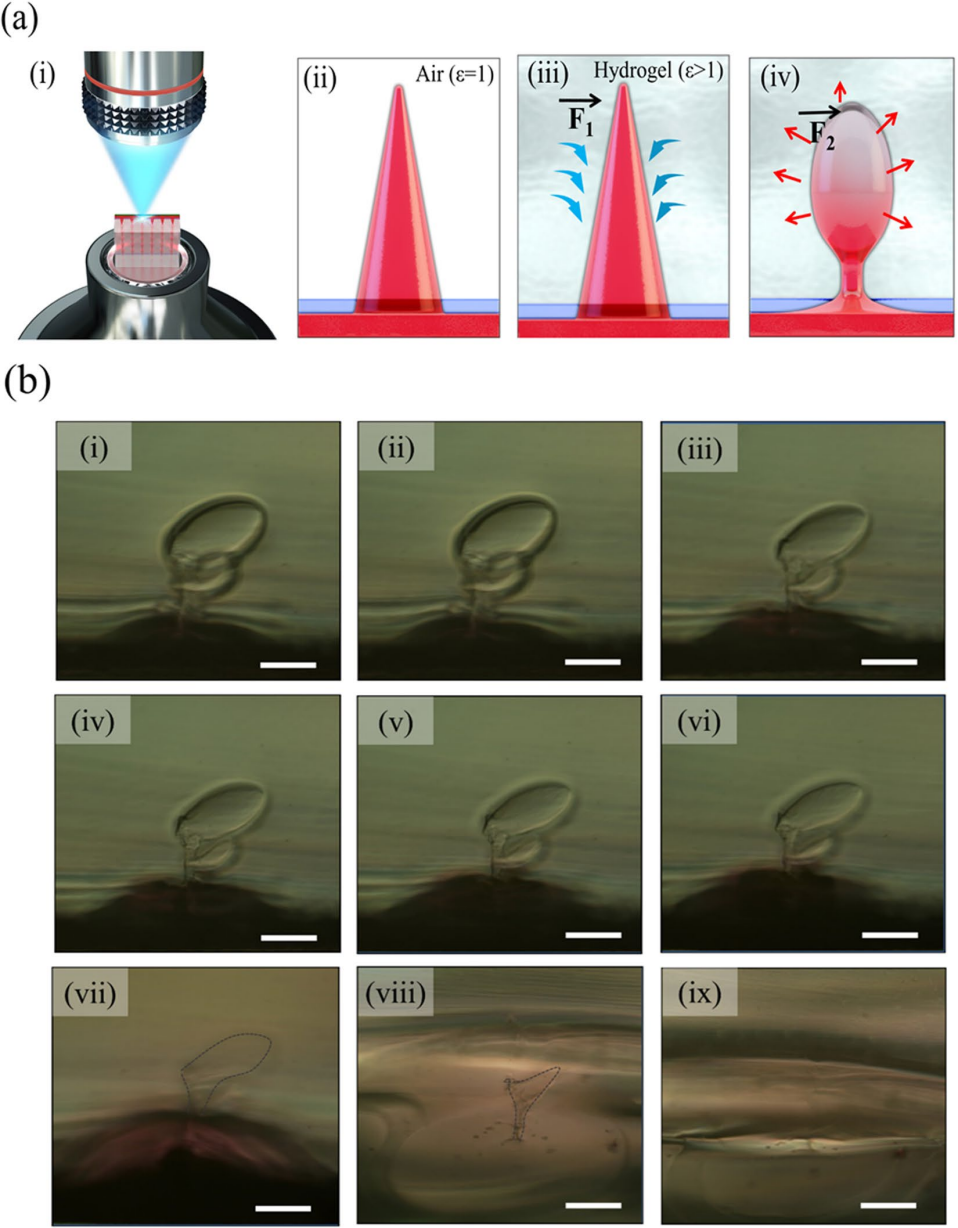
(**a**) Schematic diagrams of (i) the dissolution of the HA microneedle in gelatin hydrogel and the needle dissolution mechanism; (ii) in air (ε = 1), (iii) absorption of moisture immediately after needle insertion in hydrogel, and (iv) dissolution of swollen needle (ε > 1). (${\overrightarrow{F}}_{1}$) indicates the absorption forces and (${\overrightarrow{F}}_{2}$) indicates the dissolution forces. (**b**) Optical images of HA microneedle dissolution over time in gelatin hydrogel; (i) immediately after needle insertion in hydrogel, (ii) after 2 min, (iii) 4 min, (iv) 6 min, (v) 8 min, (vi) 10 min, (vii) 30 min, (viii) 60 min, and (ix) 140 min. Scale bar = 200 μm.

Figure 3a(i) represents the system for observing the HA microneedle dissolution phenomenon in hydrogel using an optical microscope. After the solidified hydrogel is thinly cut, microneedles containing rhodamine B penetrating the polymer membrane are inserted on the surface of the hydrogel. The penetration of the needle was in the field of view of the objective lens, and the observation magnification was fixed at 50×. The dissolution mechanism of HA microneedles in the hydrogel is shown in Fig. 3b(i–iv). When the needles were inserted, the HA microneedles absorbed and dissolved in water in the gel (Fig. 3a(iii)). Initially, the water absorption force **(**${\overrightarrow{F}}_{1}$**)**was greater than the dissolving force (${\overrightarrow{F}}_{2}$) and the needle expanded; the needle inflation gradually decreased over time (Fig. 3a(iv)). Figure 3b shows the dissolution phenomenon of HA microneedles in hydrogel over time. Immediately after insertion, the needles underwent both dissolution and swelling, but the swelling force (${\overrightarrow{F}}_{1}$) was greater than the dissolution force (${\overrightarrow{F}}_{2}$), as shown in Fig. 3b(i). After approximately 140 min, HA was fully dissolved, and rhodamine was widely spread (Fig. 3b(ix)). Figure S2 shows the images of dissolution immediately after other needles insertion and within 10 min.

### Effect of ultrasound on rhodamine release

**Figure 4:**
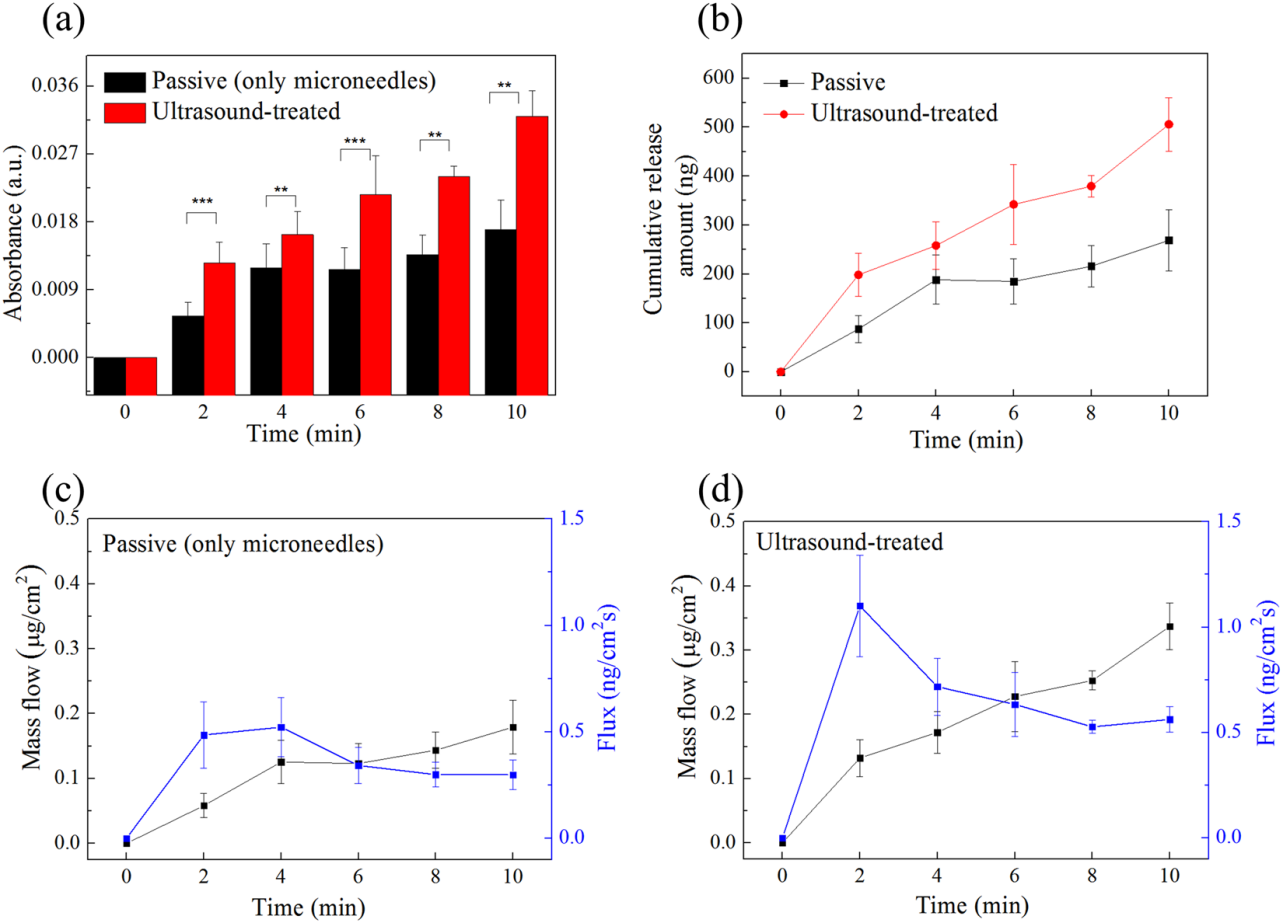
Effect of ultrasound overtime on (**a**) absorbance, *Indicates statistical significance compared to the group “passive” at the level of p values using a student’s t-test; n = 3/group. (**b**) Cumulative release amount, (**c**) mass flow and flux (passive condition), and (**d**) mass flow and flux (with ultrasound-treatment) of rhodamine B.

To accelerate the dissolution time of HA microneedles, ultrasonic waves were applied, and the effect on rhodamine release was analyzed by absorption spectrum measurement over time. Fig. S3 is the absorption spectrum for the amount of rhodamine in the needle tip. The dotted line indicates the peak point of rhodamine absorbance at ~558 nm. Figure 4a shows the absorbance measured at 2-min intervals according to the presence or absence of ultrasonic waves. Detailed experimental methods are described in the experimental section. The results shown in Fig. 4b revealed that the absorbance of rhodamine was about 250 ng and 500 ng in the presence or absence of ultrasound, respectively, for 10 min.

There are two primary factors that account for the diffusion of rhodamine B in the presence of ultrasound. First; the HA needle, vibrated by ultrasound, acts as a microfluidic channel to promote drug diffusion. Second; there may be a thermal effect caused by ultrasound, but the thermal effect is insignificant, since commercial devices are designed to be skin friendly. Therefore, the diffusion of rhodamine in the gel is the only needle vibration caused by acoustic pressure of ultrasound. Figure S4 shows the penetration depth and diffusion with and without ultrasound. The patches were peeled off after 5, and 10 min and the cross-section of the gelatin was observed. The spread of rhodamine was increased in the presence of ultrasound. In addition, as shown in Figure S4(b), the difference in relative penetration depth of rhodamine was caused by ultrasonic waves. From the transportation phenomenon perspective, the flux was calculated as shown in Fig. 4c,d for comparison between the presence (US) or absence (passive condition) of ultrasonic waves. For the calculation, an area of 1.5 cm^2^ was used as the patch area. In the passive condition, the flux increased until 4 min, then decreased slightly and became constant. In the case of US, the flux increased rapidly at 2 min, and then decreased slightly. Therefore, the dispersion of rhodamine B was the fastest within the first 2 min, and subsequently remained unchanged; however, the slope up to 2 min was larger for the microneedles treated with ultrasonication.

### Effect of iontophoresis and combination of iontophoresis and ultrasound on rhodamine release

Experiments were performed to compare the drug absorption rate when continuous sine waves were applied to the HA microneedles. AC current iontophoresis has been proposed to remove electrochemical burns from overcurrent and to reduce skin irritation that may occur in situations where inappropriate electrode designs are used^28^. For AC iontophoretic transport modeling, the interaction between the applied AC frequency and applied AC voltage on the HA microneedles was investigated. The voltage was applied to the positive electrode on the patch side and to the negative electrode on the hydrogel side. Figure S5 shows the absorption of rhodamine in response to voltage and frequency. When the voltage was applied, the rhodamine dissolved and the ions were propelled by the repulsion between the same charges, thereby facilitating the permeation of rhodamine.

**Figure 5:**
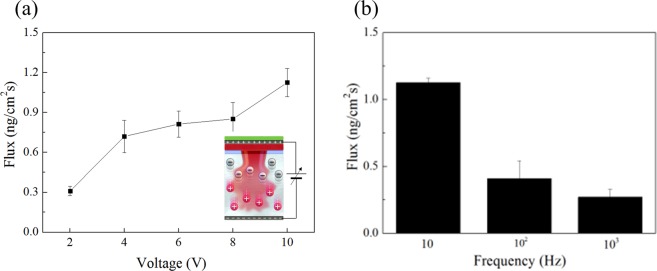
Effect of iontophoresis on (**a**) flux according to voltage and (**b**) flux according to the frequency at fixed 10 V.

The effect of iontophoresis in transdermal drug delivery, in which HA is negatively charged and rhodamine is positively charged, and polarity is alternated, can be seen in Fig. 5. As shown in Fig. S5b, the absorbance decreased with increasing frequency.

The lowest frequency required to reach maximum AC flux enhancement was 10 Hz (Fig. 5b). Therefore, as the AC frequency increased further, passive transport dominated and showed low flux of ion-permeable material. In addition, the absorbance, according to the voltage, was measured. In the case of a voltage ≥ 10 V, the maximum voltage was set at 10 V to prevent the dissolution of gelatin due to melting. The amount of the rhodamine released in 2 min increased with the increase in voltage, and the electric field at 10 V was 33. 3 V cm^−1^. Here, the electric field also plays a role in heating as well as providing the driving force to the diffused ions by electrical repulsion.

**Figure 6:**
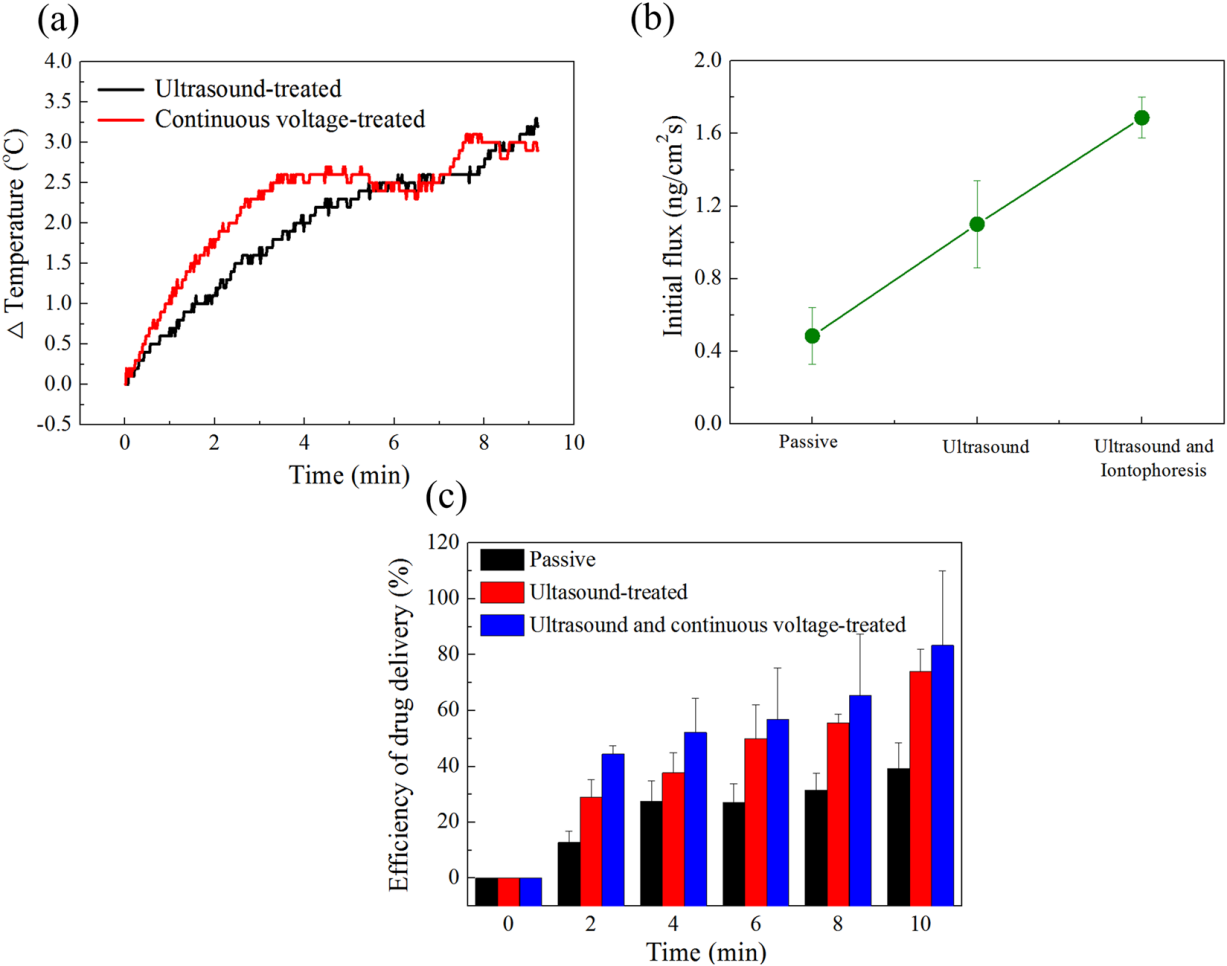
(**a**) Temperature change with time, (**b**) comparison of initial flux at 2 min, and (**c**) drug delivery efficiency over time.

Heat generation was investigated to determine energy transfer to ultrasonic and current. The temperature using thermometer inserted between the microneedle array and gel. The starting hydrogel surface temperature was about 19 °C, and it was not fixed at 37 °C due to the gelatin gelation process and thermos-reversibility^41^. Here, the temperature change is important, not the initial gel surface temperature. The surface temperature of the gel varies with the presence of ultrasonic wave and current, and the results are shown in Fig. 6a. The change in temperature was about ~3 °C for 10 minutes for ultrasound, and the change in temperature by ultrasound is related to their intensity^13,23^, which was set to be constant in our experiment. The change in temperature due to the current was about ~4 °C for 10 minutes, which is evidence for heat generation by electric current absorption in gelatin membrane.

Figure 6b,c compares drug efficiency to see the synergy of ultrasonic and continuous current. Figure S6 shows the measured absorbance and flux of HA microneedles when combined with ultrasonic waves and AC electric field, with time dependence. The greatest increase in flux occurred at the initial 2 min, and when combined with ultrasonic waves and electrical stimulation, further increase was observed (Fig. 6b).

The combined efficiency of ultrasound and electric field treatment for 10 min increased from about 39.3% to about 83.4%, compared to the passive condition (Fig. 6c). Thus, the synergy of ultrasonic waves and electric field improves drug delivery efficiency. Dissolution is promoted by the vibration caused by the ultrasonic waves and the propulsion caused by the electrostatic force. Therefore, our study suggests that treatment with ultrasonic waves and iontophoresis in combination can increase the drug delivery rate.

In conclusion, we established a high-performance drug delivery system using HA microneedles in combination with ultrasound and AC ionic field. Here, the HA microneedles were fabricated using the concentrations of different HA solution, and the needle shape was compared. Our results showed that the filling amount of HA in the tip could be controlled by adjusting the HA concentration. The manufactured HA microneedles can be inserted into hydrogel or skin to form pores and dissolved for the drug delivery. Ultrasonic and AC ionic fields were used to increase the rate of drug absorption. The function of the ultrasonic wave is to vibrate the needles, and the continuous electric field uses the electrostatic force to promote the permeation of the drug. To demonstrate the efficacy of each approach, release experiments were performed *in vitro* using a gelatin hydrogel, and fluxes were calculated. In the initial 2 min (in passive), the flux increased, then decreased, and became constant. When the ultrasonic wave and electric field were combined, the slope of the initial flux increased than the passive. Thus, ultrasound and electrical stimulation had a significant effect on drug release during the initial stage. By combining these multiple strategies, the drug delivery efficiency was improved. Thus, these microneedles platform with ultrasonic-assisted iontophoresis provides an alternative to rapid drug response compared to a drug delivery system based on passive diffusion.

## Methods

### Fabrication of HA solution

HA was dissolved in de-ionized water (DI water) to prepare the HA solution (hydrolyzed sodium hyaluronate, Bloomage Freda Biopharm co. Ltd., molecular weight: 200~400 kDa). The final concentrations of the HA solution used in the study were 1.0, 2.0, 3.0, and 4.0 wt%. Magnetic stirring at approximately 550 rpm at room temperature for one day, was required to obtain a homogeneous mixture of HA and DI water.

### Fabrication of the microneedle mold

Commercial Derma stamp (Larcobaleno, Seoul, South Korea) was used as a master stamp for microneedle array production. The microneedles were designed with a height of 1000 µm (base width of 2000 µm and tip radius of 10 µm). PDMS (Sylgard 184; Dow Corning, Midland, MI, USA) prepared by mixing elastomer and curing agent in a ratio of 10: 1 was poured onto a master stamp. The molds were cured at 50 °C for 2 h. The PDMS mold was then separated from the stamp^10,11,42^.

### Fabrication of microneedles

Rhodamine B (20 mg; >98% pure; Acros-Organics, Geel, Belgium), a water-soluble red fluorescent dye, was mixed in the HA solution. Subsequently, the HA solution (1 mL) mixed with rhodamine B was cast over the PDMS mold, and the mold was vacuum-treated for 5 min. Then, the remaining solution (1.5 mL) was cast and dried in an oven (ThermoStable ON-105; DAIHAN Scientific) for 6 h. After solidification, the HA microneedle patch was separated from the PDMS mold.

### Ultrasound stimulation

Ultrasonic functions comprised 1.7 million micro-vibrations per second. The ultrasound gel (Ecosonic, SANIPIA, Republic of Korea) was applied between the transducer and gelatin to improve acoustic impedance matching.

### Electrical stimulation

For the electrode in the electrical stimulation experiment, the *in vitro* IP system consisted of a sine wave pulse generator and a caliper electrode. The caliper electrode consisted of two plate electrodes, one with a silver paste on the microneedle patch backplate and the other with an aluminum foil under the gel. The positive electrode was always applied to the microneedle patch. The gap (3 mm) between the two electrodes was filled with gel.

### *In vitro* drug release

Gelatin hydrogel (10 wt%; Sigma Aldrich, gel strength ~300 g Bloom, Type A) derived from porcine skin was used as the model tissue for the drug release studies^11,43^. The polymer membrane (Parafilm M, American National Can, Chicago, IL) was pierced with microneedles and placed on the hydrogel surface. At each timestamp, the microneedle patch was detached from the hydrogel to stop drug release. To separate the drug and the drug-diffused gelatin, the gel was dipped in a decolorizing solution (methanol:DI:acetic acid, 5:4:0.5) for ~6 h. The absorbance of the decolorized gelatin gel solution was measured.

### Statistical analysis

All statistical data were analyzed by Student’s t-test for statistical significance.

## Supplementary information

Supplementary Information.

## Data Availability

The datasets generated during and/or analyzed during the current study are available from the corresponding author on reasonable request.
