# Supplementary material for: Ultrasonically and Iontophoretically Enhanced Drug-Delivery System Based on Dissolving Microneedle Patches
Source: Sci Rep. 2020 Feb 6;10:2027. doi: 10.1038/s41598-020-58822-w (PMC7005184; doi:10.1038/s41598-020-58822-w)
Supplement: Supplementary file 1 — Supplementary Information. [file 41598_2020_58822_MOESM1_ESM.docx]

Supporting Information

Ultrasonically and Iontophoretically Enhanced Drug-Delivery System Based on Dissolving Microneedle Patches

Moonjeong Bok^1,2^, Zhi-Jun Zhao^2^, Sohee Jeon^2^, Jun-Ho Jeong^2,3*^, Eunju Lim^1*^

^1^ Department of Science Education/Creative Convergent Manufacturing Engineering, Dankook University, Yongin 16890, Korea

^2^ Nano-Convergence Mechanical Systems Research Division, Korea Institute of Machinery and Materials, Daejeon 34103, Korea

^3^ Department of Nano Mechatronics, University of Science and Technology, Daejeon 34103, Korea

* Corresponding authors: elim@dankook.ac.kr; jhjeong@kimm.re.kr


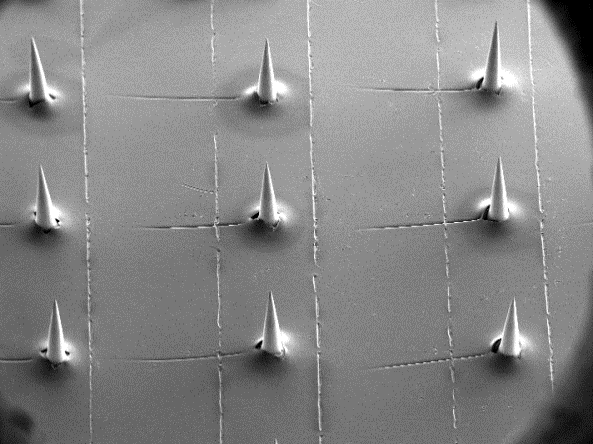


**Figure S1.** Scanning electron microscopy image of the hyaluronic acid (HA) microneedle array. Scale bar: 1000 μm.


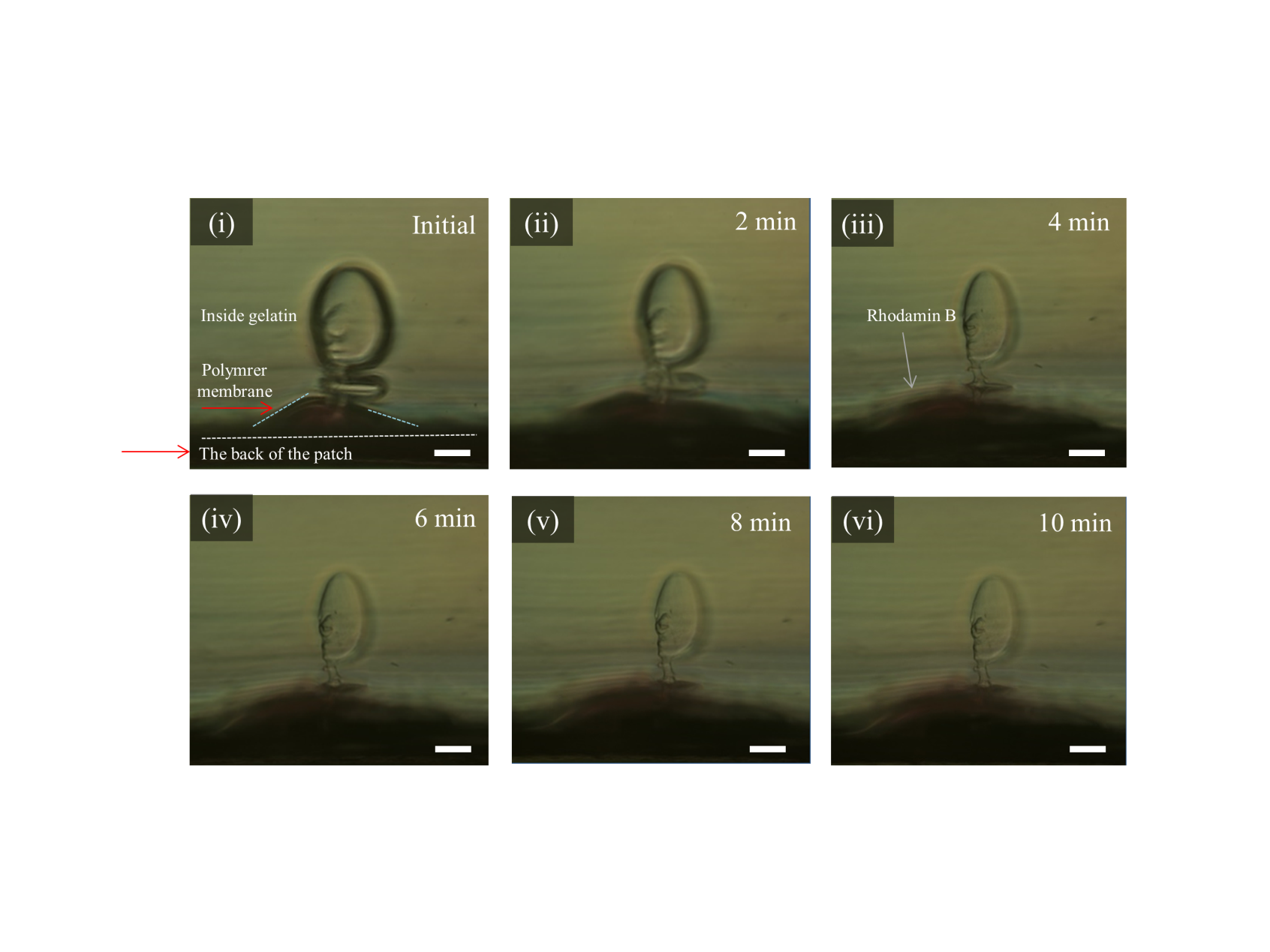


**Figure S2.** Optical images of HA microneedle dissolution over time in the gelatin hydrogel; (i) immediately after needle insertion in the hydrogel and (ii) after 2 min, (iii) 4 min, (iv) 6 min, (v) 8 min, and (vi) 10 min. Scale bar = 100 μm.


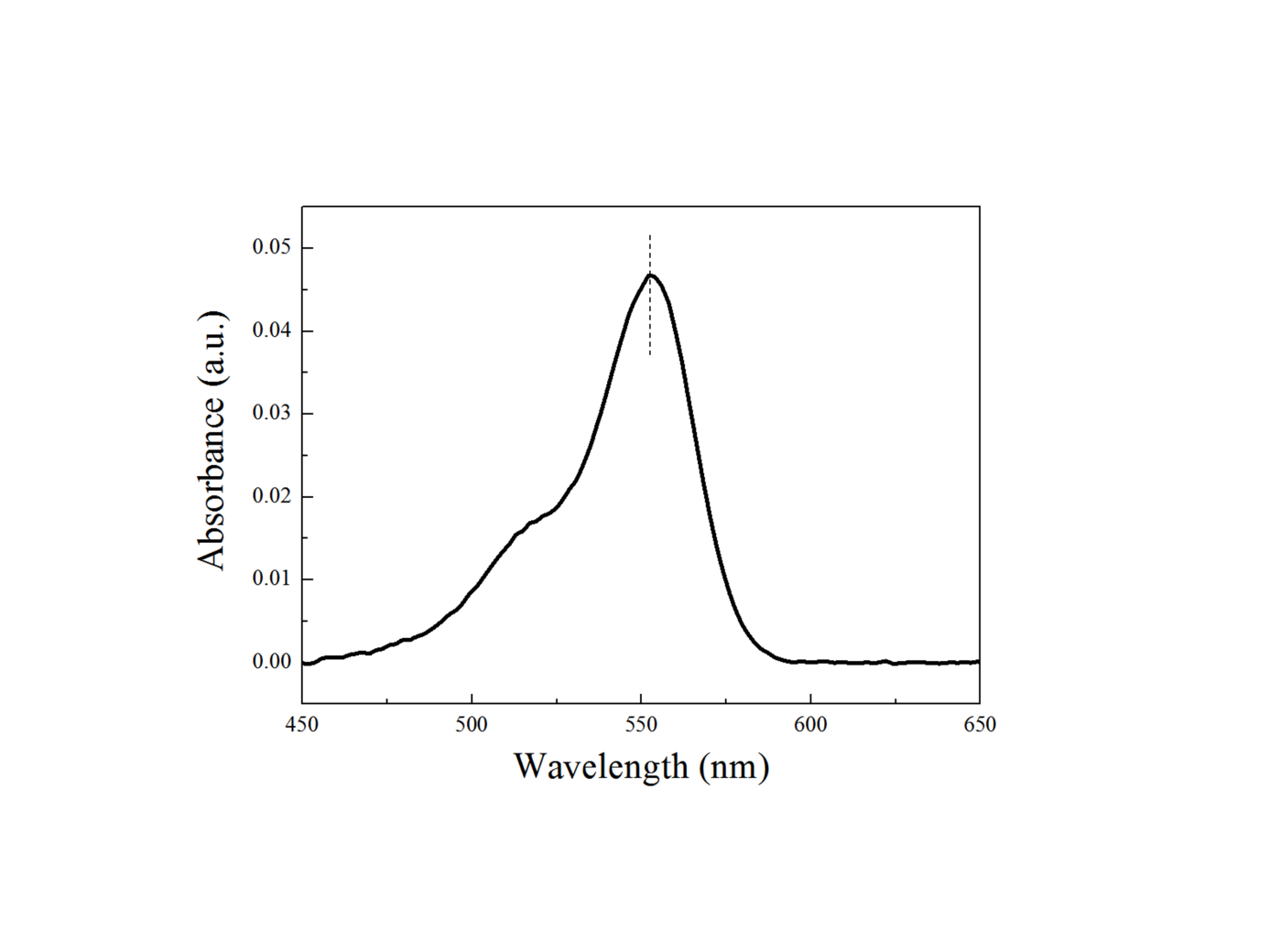


Figure S3. Rhodamine B absorbance at the needle tip. The dotted line indicates the peak at ~558 nm.


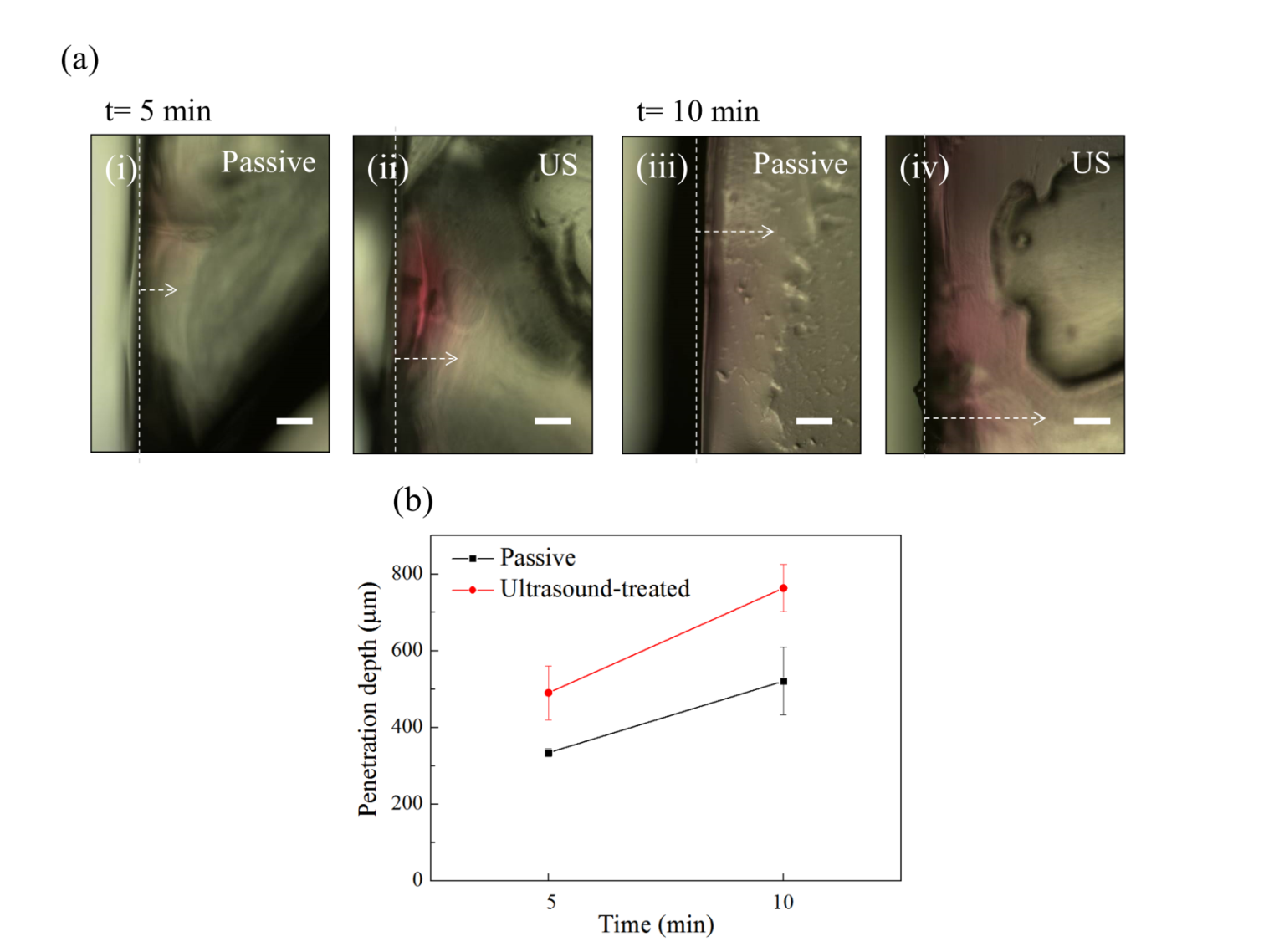


Figure S4. (a) Cross-section of gelatin without (passive) and with ultrasound (US) treated penetration with HA microneedle; (i-ii) after 5 min, (iii-iv) and 10 min; (b) the difference in penetration depth. Scale bar = 250 μm.


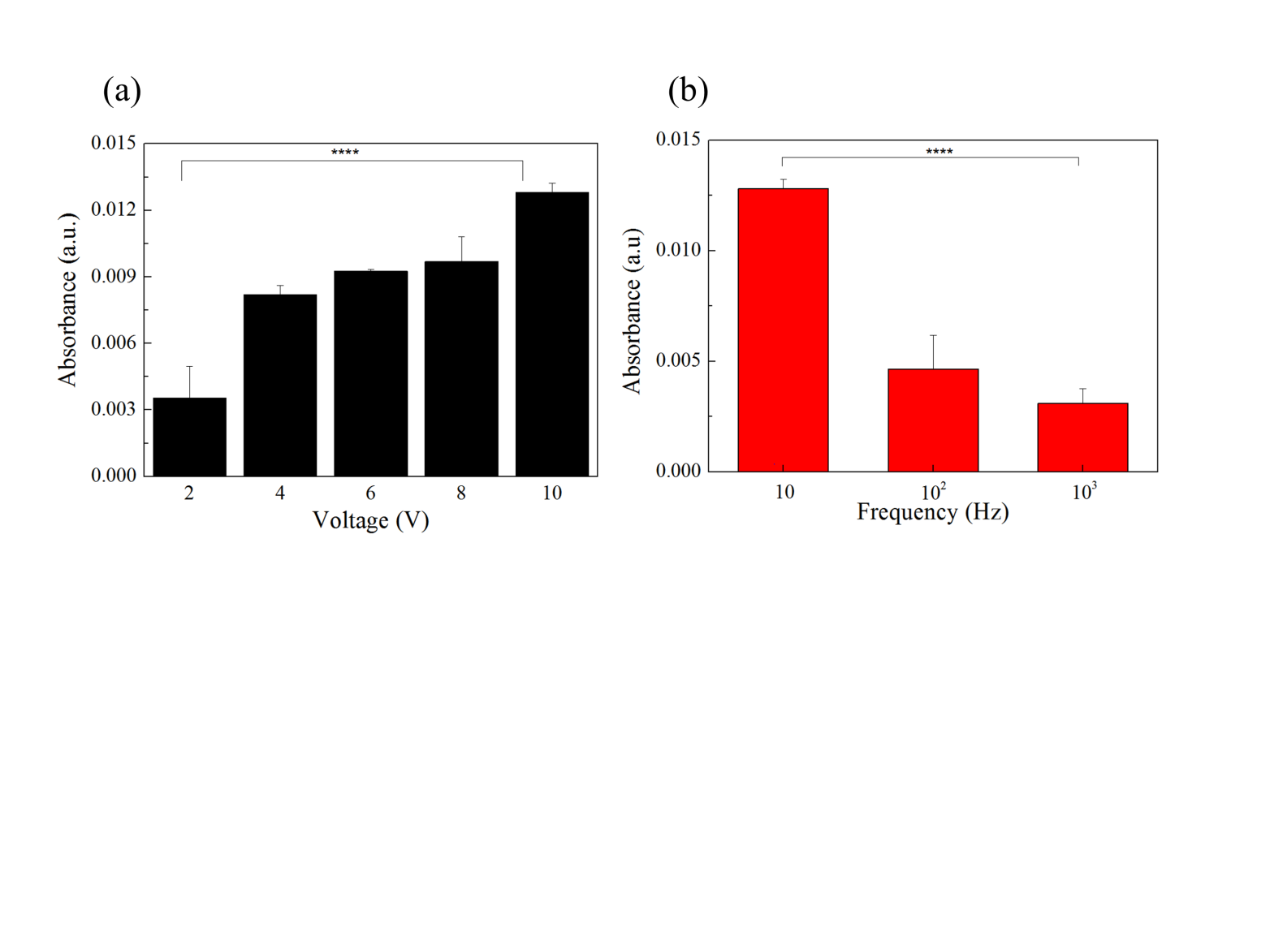


Figure S5. (a) Absorbance with the voltage (n = 3, ****p < 0.0001) and (b) absorbance with the frequency for 2 min (n = 3, ****p < 0.0001).


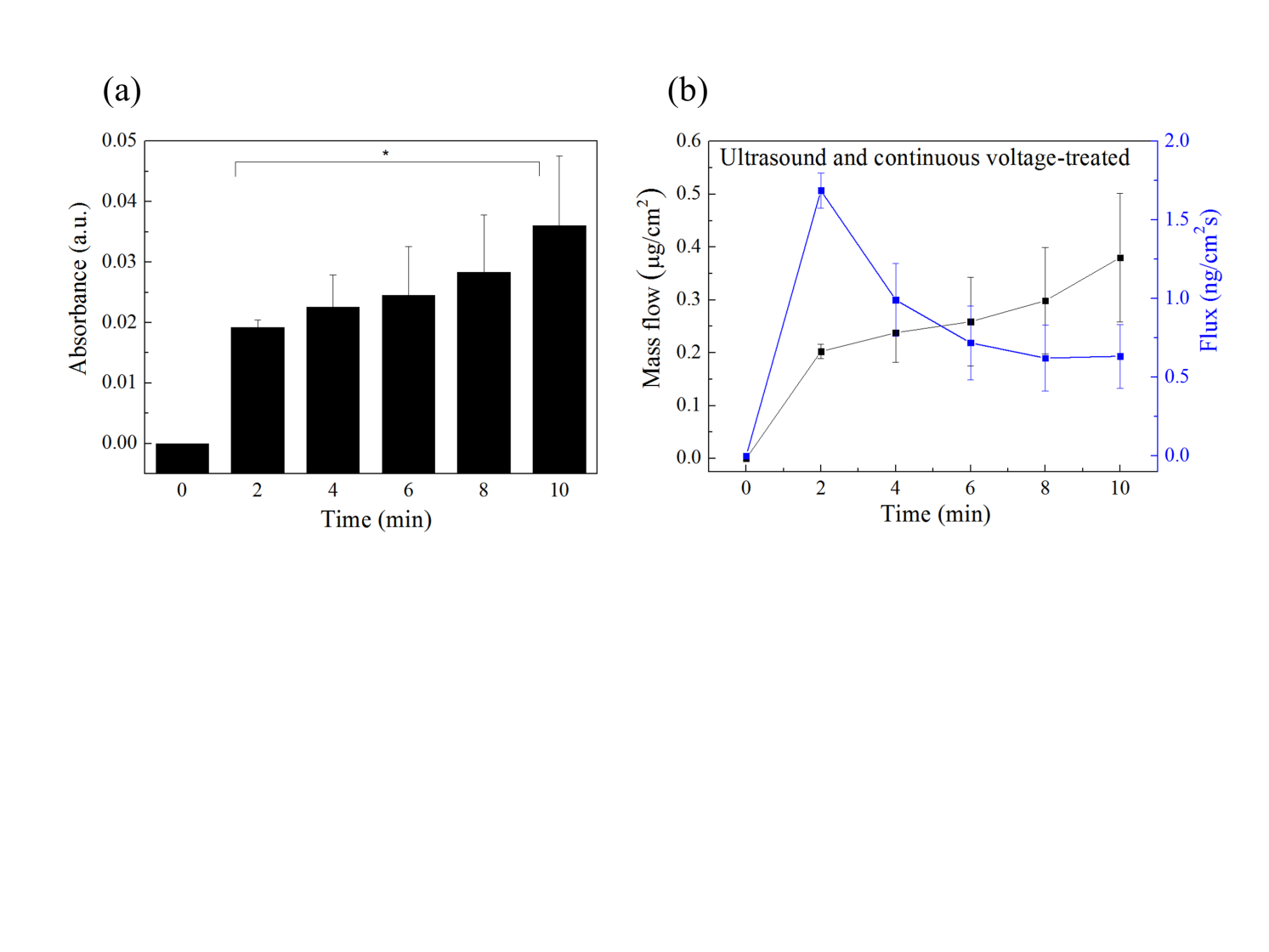


Figure S6. (a) Absorbance over time (n = 3, *p < 0.05) and (b) mass flow and flux overtime when ultrasonic waves and continuous voltage were applied.
